# Unveiling the Therapeutic Potential of *Piper chaba* Hunter: Computational Approaches Shed Light on Targeting Proteins in Alzheimer's Disease

**DOI:** 10.1155/bmri/8892801

**Published:** 2025-07-07

**Authors:** Md. Sifat Rahi, Md. Shahedur Rahman, Rima Islam Meem, Md. Ebrahim Khalil Shimul, Nahid Farnaj, Md. Humaun Kabir, Fee Faysal Ahmed, Md. Anowar Khasru Parvez, Md. Amdadul Huq, Tabassum Kabir, Abdel Halim Harrath, Mahadi Hasan, Md. Ataur Rahman

**Affiliations:** ^1^Department of Genetic Engineering and Biotechnology, Jashore University of Science and Technology, Jashore, Bangladesh; ^2^Bioinformatics and Microbial Biotechnology Laboratory, Department of Genetic Engineering and Biotechnology, Jashore University of Science and Technology, Jashore, Bangladesh; ^3^Department of Physics, Jashore University of Science and Technology, Jashore, Bangladesh; ^4^Department of Mathematics, Jashore University of Science and Technology, Jashore, Bangladesh; ^5^Department of Microbiology, Jahangirnagar University, Savar, Bangladesh; ^6^Department of Life Sciences, College of BioNano Technology, Gachon University, Seongnam, Republic of Korea; ^7^M Abdur Rahim Medical College Hospital, Dinajpur, Bangladesh; ^8^Department of Zoology, College of Science, King Saud University, Riyadh, Saudi Arabia; ^9^Global Biotechnology & Biomedical Research Network (GBBRN), Department of Biotechnology and Genetic Engineering, Faculty of Biological Sciences, Islamic University, Kushtia, Bangladesh

**Keywords:** Alzheimer's disease, metabolic pathway, network pharmacology, *Piper chaba*, PLA2G4A protein, PTGS2 protein

## Abstract

Alzheimer's disease (AD) is a prevalent neurodegenerative disorder, while the existing treatments primarily focus on alleviating symptoms rather than addressing the underlying pathophysiology. Seeking a safer alternative, the study explores the potential of *Piper chaba* Hunter as a promising drug lead for AD by eliciting the major signaling pathway, key players, and their interaction with phytochemicals from the plant extract. Initially, the phytochemicals in the *P. chaba* crude extract were identified using GC-MS, and their physicochemical properties were verified using SwissADME. Protein–protein interaction (PPI) and signaling pathways–target proteins–compounds (STC) networks were analyzed to dig out target proteins and effective compounds for AD based on rigorous screening. Approximately 60 target proteins that interacted with GC-MS-identified compounds underwent PPI and STC networking which identified five compounds, a signaling pathway, and three target proteins with therapeutic potential. Three compounds, namely, bicyclo[7.2.0]undec-4-ene, 4,11,11-trimethyl-8-methylene-,[1R-(1R∗,4Z,9S∗)], 2-methoxybenzoic acid, 2,3-dichlorophenyl ester, and (E)-3-butylidene-4,5-dihydroisobenzofuran-1(3H)-one, have the potential to modulate PTGS2, PLA2G4A, and CYP2C19 within metabolic signaling pathway, thus serving as promising therapeutic agents. Moreover, the drug likeliness and efficacy of those phytochemicals were justified by molecular docking tests (MDTs), molecular dynamics simulations (MDSs), and quantum chemistry analyses, which confirmed their ability to inhibit key targets to mitigate AD-associated pathology.

## 1. Introduction

Alzheimer's disease (AD) is a debilitating brain cell degeneration disorder characterized by dementia and cognitive dysfunction, ultimately leading to death [1]. The formation of alpha-amyloid plaques and neurofibrillary tangles consisting of hyperphosphorylated tau is considered key indicators of AD [2]. Mitochondrial dysfunction resulting from an imbalance in cellular bioenergetics has also been implicated in the pathogenesis of AD [3, 4]. However, the etiology of the sporadic form of the disease remains unclear, and existing treatment strategies, such as donepezil and metrifonate, are associated with ineffective outcomes and adverse effects [5].

Current AD treatments, such as cholinesterase inhibitors (e.g., donepezil) and NMDA receptor antagonists (e.g., memantine), primarily offer symptomatic relief without altering disease progression. Notably, a meta-analysis concluded that memantine was ineffective for patients with mild AD, and its benefits for moderate cases were inconsistent [6]. Additionally, numerous therapeutic trials targeting amyloid-beta (A*β*) accumulation have failed to demonstrate clinical efficacy, casting doubt on the amyloid hypothesis as a singular therapeutic target [7]. These treatments are also associated with adverse effects, including gastrointestinal disturbances, dizziness, and headaches, which can impact patient compliance and quality of life [6]. These limitations underscore the urgent need for novel therapeutic approaches that can effectively modify disease progression while minimizing adverse effects.

To explore alternative treatment options, researchers have turned their attention to medicinal plants, which offer a rich source of bioactive compounds, including lignin, flavonoids, tannins, polyphenols, triterpenes, sterols, and alkaloids [8]. These phytochemicals have demonstrated diverse pharmacological activities, such as anti-inflammatory, antiamyloidogenic, anticholinesterase, and antioxidant effects [9–12]. Importantly, the nontoxic nature and minimal adverse effects associated with phytochemicals make them attractive candidates for AD treatment [13, 14]. Consequently, researchers worldwide are actively investigating the potential of medicinal plants as sources of highly effective drug leads for AD [15].

Bangladesh, as a tropical country, boasts a wealth of indigenous medicinal plants with known and unknown pharmacological properties. Among these, *Piper chaba* Hunter (Piperaceae) is widely used as a spice in the southern region of Bangladesh, particularly in certain districts of the Khulna Division [16]. Previous studies have reported various pharmacological activities, including anti-inflammatory, antihyperlipidemic, antidepressant, immunoregulatory, analgesic, and antipyretic effects associated with different parts of *P. chaba* [16–19]. Consequently, it is crucial to assess the potential of phytochemicals present in *P. chaba* as natural drug candidates against AD.

**Figure 1:**
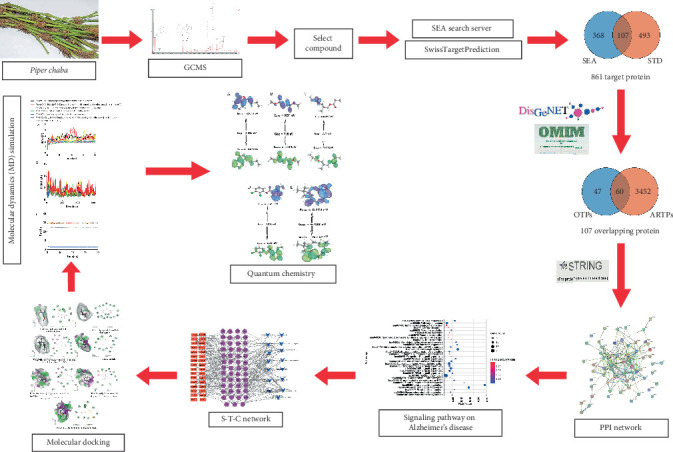
Network pharmacology analysis workflow of *P. chaba* phytochemicals against AD.

Andrew Hopkins' network pharmacology concept, based on bioinformatics and systemic biology, provides a promising approach for studying the interactions between disease-related genes, proteins, and target compounds [20–22]. This well-established strategy allows for the identification of bioactive plant-based compounds and their underlying mode of actions against various diseases [23]. Moreover, network pharmacology facilitates the elucidation of the complex links between the pharmacological activities of specific molecules and the biochemical pathways associated with particular diseases [24, 25]. Schematic workflow of network pharmacology analysis of *P. chaba*–derived compounds in AD therapy is presented in Figure 1.

Therefore, the present study is aimed at evaluating the bioactive compounds from *P. chaba* using gas chromatography–mass spectrometry (GC-MS) analysis and investigating their mechanisms of action against AD through the application of network pharmacology. Additionally, an in silico analysis will explore the interactions between phytochemicals and the genes involved in AD progression, providing valuable insights into the most promising drug candidates derived from *P. chaba* for the treatment of AD.

## 2. Methods and Materials

### 2.1. Plant Collection and Extraction Procedure

*P. chaba* plant was collected from the Jashore District of Bangladesh. The collected plant parts were rinsed with distilled water and cut into little pieces. They were then allowed to air dry for 15 days at room temperature. The plant parts were ground to a fine powder using the grinder. The powder was then utilized to make a methanolic extract (99% methanol) by following the previously described protocol by Islam et al. [26].

### 2.2. GC-MS Analysis

GC-MS analysis was carried out at the “Centre for Sophisticated Instrumentation and Research Laboratory (CSIRL), Jashore University of Science and Technology, Bangladesh.” The analysis was performed using a Clarus 690 gas chromatograph (PerkinElmer, CA, United States) fitted with an Elite-35 column (30 m length, 0.25 mm diameter, and 0.25 *μ*m film thickness) and connected to a Clarus SQ 8 C mass spectrometer (PerkinElmer, CA, United States). A 1 *μ*L sample extract was injected in splitless mode, with helium (99.999%) as the carrier gas at a steady flow rate of 1.0 mL/min. The GC-MS interface was maintained at 280°C, ensuring optimal transfer between the systems. The oven temperature program started at 60°C, held for 0 min, then increased at a rate of 5°C per minute until reaching 240°C, where it was held for 4 min. The total runtime for the analysis was 40 min. For mass detection, the spectrometer operated in electron ionization (EI) mode at 70 eV. The quadrupole and ion source temperatures were set at 150°C and 230°C, respectively. The instrument scanned masses ranging from 50 to 550 m/z. Compound identification was done by comparing the mass spectra with the National Institute of Standards and Technology (NIST) database, and the relative abundance of each compound was determined based on peak area [27].

### 2.3. Property Screening of Phytochemicals

Absorption, distribution, metabolism, and excretion (ADME) properties of the isolated compounds were assessed using computational tools, specifically SwissADME (http://www.swissadme.ch/, accessed on 8th June 2022) and PubChem (https://pubchem.ncbi.nlm.nih.gov/, accessed on 8th June 2022) [28, 29]. The compounds' Simplified Molecular Input Line Entry System (SMILES) formats were obtained for subsequent analysis. SwissADME is a well-established online tool widely employed for calculating essential physicochemical, pharmacokinetic, drug-like, and related parameters of compounds [2]. Conversely, PubChem serves as a comprehensive repository of chemical structures, bioactivity, health and safety data, spectra data, and other relevant information, offering direct data access through programmatic services and FTP downloads [3].

### 2.4. Compound, Target Protein, and Pathway Analysis

The similarity ensemble approach (SEA) database (http://sea.bkslab.org/, accessed on 11 June 2022) and SwissTargetPrediction (STP) database (http://www.swisstargetprediction.ch/, accessed on 12 June 2022) were employed to identify target proteins associated with the detected phytochemicals using GC-MS analysis [30, 31]. Only human-specific targets were considered, and duplicate entries were removed. Target proteins associated with AD were screened from the Online Mendelian Inheritance in Man (OMIM) database (https://www.omim.org, accessed on 13 June 2022) and the DisGeNET database (https://www.disgenet.org/search, accessed on 13 June 2022) [32, 33]. Venn diagrams were subsequently constructed to identify the common target proteins by illustrating the interaction between phytochemicals and target proteins, as well as phytochemicals and AD-associated target proteins.

Multiple databases were used to ensure data robustness, cross-validation, and comprehensive target coverage, thereby minimizing database-specific biases. The rationale behind combining SEA and STP was to enhance both structure-based and ligand-based prediction accuracy. Similarly, integrating OMIM and DisGeNET allowed us to capture both curated and literature-mined disease associations.

Furthermore, signaling pathways relevant to AD were collected through functional enrichment analysis utilizing the STRING database (https://string-db.org/, accessed on 16 June 2022), with a confidence score cutoff of ≥ 0.4. Kyoto Encyclopedia of Genes and Genomes (KEGG) and GO pathway enrichment were performed with a statistical significance threshold of *p* < 0.05 [34]. The signaling pathways–target proteins–compounds (STC) network analysis facilitated the construction of a network pathway based on the significance of the target proteins. All the gathered data was prepared and analyzed using Cytoscape software. The screening of target proteins was conducted using the default parameters of the respective databases, and multiple databases were used to enhance reliability and minimize biases.

### 2.5. Molecular Docking Study

The proteins of interest were obtained in .pdb format from the RCSB Protein Data Bank (https://www.rcsb.org/, accessed on 4 July 2022) database and subsequently prepared for molecular docking analysis using Discovery Studio software [2]. The protein was prepared by removing water, bound complex molecules, heteroatoms, and additional chains of protein. The docking procedure was performed by utilizing PyRx software employing the AutoDock Vina module, which involved the docking of the selected proteins with the corresponding compounds [35]. The specific docking sites on the proteins are detailed in Table 1.

### 2.6. Molecular Dynamics Simulation (MDS)

The MDS investigation involving the selected ligand–receptor interactions was performed employing the YASARA structure and WHAT IF software package (Version 22.5.22) [2, 36]. The AMBER14 force field and TIP3P solvation model were utilized within a cubic simulation cell featuring a periodic boundary condition [37, 38]. To simulate physiological conditions, the temperature was set at 298 K, pH at 7.4, and the concentration of NaCl at 0.9% in the simulation cells, where solvent density was maintained at 0.997 g/L. The hydrogen bond network was optimized to ensure the correct protonation states under physiological conditions using the SCWRL. The system temperature and energy were stabilized through an equilibration phase of approximately 1.5 ns.

Prior to the simulation, an energy minimization procedure was conducted using an energy-simulated annealing method assisted by the steepest gradient algorithms, comprising 5000 cycles. The simulation setup incorporated a constant pressure and Berendsen thermostat [39]. Long-range electrostatic interactions were computed using the particle mesh Ewald (PME) method, with a cutoff radius set at 8 Å [40]. Subsequently, the simulation was carried out for a duration of 20 ns, during which trajectories were generated to assess various parameters such as root mean square deviations (RMSDs), root mean square fluctuations (RMSFs), and the radius of gyration (Rg). These parameters were employed to evaluate the structural stability and dynamic behavior of the ligand–receptor complexes. Based on the 20 ns simulation result, the PTGS2 + bicyclo[7.2.0]undec-4-ene, 4,11,11-trimethyl-8-methylene-,[1R-(1R∗,4Z,9S∗)] complex and its control PTGS2 + aspirin complex were extended for a 100 ns simulation to assess the stability of the complex.

### 2.7. Quantum Chemistry of Key Ingredients

The determination of the energetically favorable conformation and optimization of molecular structures play a crucial role in predicting binding affinity, which is contingent upon both solution phase and gas-phase energy considerations. In this study, the protein's energy was minimized using the steepest descent approach and the universal force field (UFF) method. Geometric optimizations of the ligands were performed using the Avogadro software [41]. The optimized molecular configurations were then subjected to calculations of highest occupied molecular orbital (HOMO) and lowest occupied molecular orbital (LUMO) energies, all based on the same theoretical framework.

To handle the electronic structure calculations, density functional theory (DFT) was employed, utilizing the B3LYP (Becke exchange functional, combined with the Lee, Yang, and Parrs (LYP) correlation functional) along with the 6-31G(d, p) basis sets. The ORCA program system was used to calculate DFT [42]. The HOMO and LUMO energies, as well as the energy gap between them, were determined through DFT computations. The hardness (*η*) and softness (*s*) of the compounds were evaluated using the Parr and Pearson explanation equation and the Koopmans' theorem equation, respectively [43, 44]. The hardness (*η*) and softness (*s*) can be calculated using the following formulas:

(1) $$\begin{array}{c} \eta =\frac{\left(I-A\right)}{2}, \end{array}$$

(2) $$\begin{array}{c} S=\frac{1}{\eta } \end{array}$$

where *I* = the ionization potential (−*E*_HOMO_) and *A* = the electron affinity (−*E*_LUMO_).

## 3. Result

### 3.1. Phytochemical Constituents of *P. chaba*

**Figure 2:**
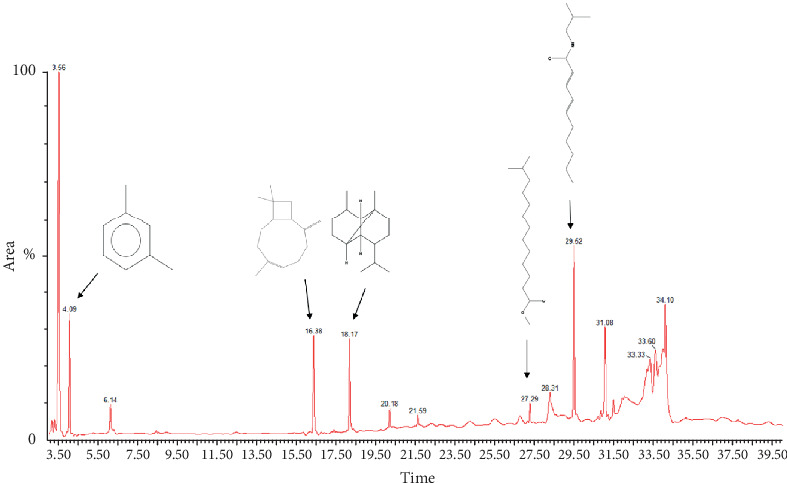
GC-MS chromatogram of *P. chaba* methanolic extract.

A total of 17 compounds were identified from the crude methanolic extract of *P. chaba* using GC-MS analysis. The chromatogram depicting the GC-MS analysis can be observed in Figure 2. However, subsequent analysis led to the selection of 15 compounds, which are presented in Table 2 along with their PubChem IDs, retention times (minutes), peak areas (%), and molecular formulas. Among the selected compounds, certain compounds were identified as major components, namely, benzene, 1,3-dimethyl-; 2-methoxybenzoic acid, 2,3-dichlorophenyl ester; 1,3-propanediol, 2-methyl-, dipropanoate; 2(3H)-furanone, 3,4-bis(1,3-benzodioxol-5-ylmethyl)dihydro-, (3R-trans)-; methyl 9-cis,11-trans-octadecadienoate; 13-octadecenoic acid, methyl ester; 2,4-decadienamide, *N*-isobutyl-, (E,E)-; and *N*-hexadecanoic acid. The remaining compounds were classified as minor components.

Initially, out of the 17 chemical compounds, 15 compounds fulfilled Lipinski's rules, including criteria such as Moriguchi octanol–water partition coefficient ≤ 4.15, molecular weight < 500 g/mol, number of nitrogen or oxygen atoms ≤ 10, and number of hydrogen bond donors or acceptors < 5. Additionally, these compounds satisfied the requirement of an “Abbott bioavailability score (> 0.1)” as per SwissADME. However, lactose was excluded due to the presence of nitrogen, oxygen, and NH or OH groups. Furthermore, the topological polar surface area (TPSA) values of the 15 chemical compounds, excluding lactose, were within an acceptable range (Table 3).

### 3.2. Common Assembled Target Proteins Between SEA and STP

The SEA and STP methodologies yielded a total of 475 and 700 target proteins, respectively, which were associated with the 15 chemical compounds derived from the plant extract. A Venn diagram was employed to visualize the overlap, revealing a shared set of 107 target proteins.

### 3.3. Common Assembled Target Proteins Between AD-Related Target Proteins and 107 Target Proteins

**Figure 3:**
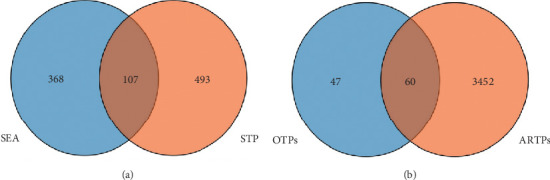
Assessment of common target proteins from databases. (a) One hundred seven overlapped target proteins between SEA and STP databases. (b) Sixty overlapped final target proteins from (OTPs) (107) and AD-related target proteins (ARTPs) (3512).

In addition, a comprehensive search of the DisGeNET and OMIM databases resulted in the identification of 3512 target proteins associated with AD. Notably, among the 107 target proteins identified in the overlap analysis, 60 proteins were found to be common to both the overlapping targets and the AD-related target proteins, as visualized in Figure 3b using a Venn diagram.

### 3.4. PPI Networking

**Figure 4:**
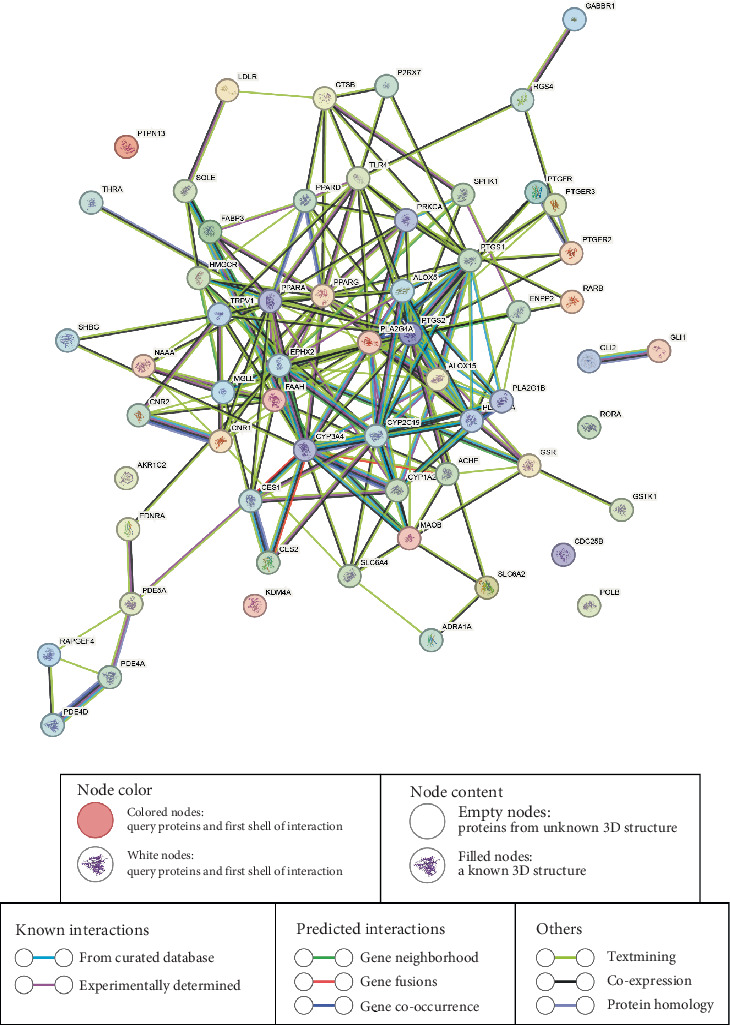
Overview of PPI networks (60 nodes and 159 edges).

The PPI of the final 60 overlapping target proteins (OTPs) was investigated using the STRING server, which generated a network comprising 60 nodes and 159 edges (Figure 4). Notably, six proteins, namely, AKR1C2, KDM4A, POLB, RORA, SHBG, and PTPN13, did not exhibit any interactions with other proteins within the network. Analysis of the PPI network revealed that PTGS2 exhibited the highest degree of connectivity, with 22 interacting partners. Following PTGS2, other proteins such as PPARA (19), PPARG (18), PLA2G4A (16), and CYP2C19 (15) displayed notable degrees of connectivity (Table 4). Based on these findings, PTGS2 emerged as the top target protein within the PPI network.

### 3.5. Analysis of Signaling Pathways Against AD

**Figure 5:**
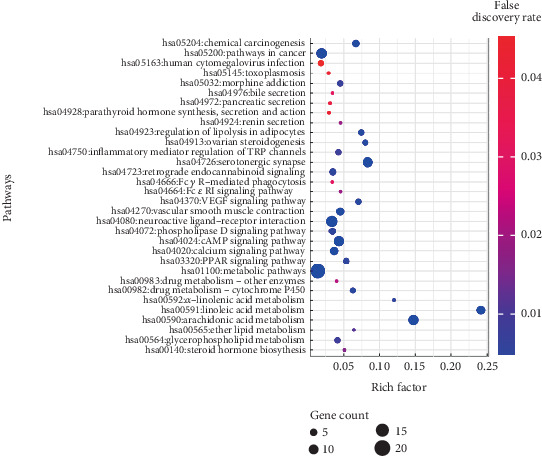
Bubble chart of *P. chaba* against AD.

The pathway enrichment analysis using KEGG revealed that among the 60 OTPs, 51 proteins were found to be associated with 32 signaling pathways directly linked to AD, with a false discovery rate of 0.05. Detailed descriptions of these pathways can be found in Table 5. Notably, the metabolic signaling pathway displayed a significant enrichment, as indicated by a rich factor of 0.024 in the bubble charts (Figure 5). A pathway enrichment threshold of 0.05 was employed to identify enriched pathways. Further analysis supported the association of the PTGS2 protein, which exhibited the highest degree of connectivity, with the metabolic signaling pathway.

### 3.6. STC Network Analysis

**Figure 6:**
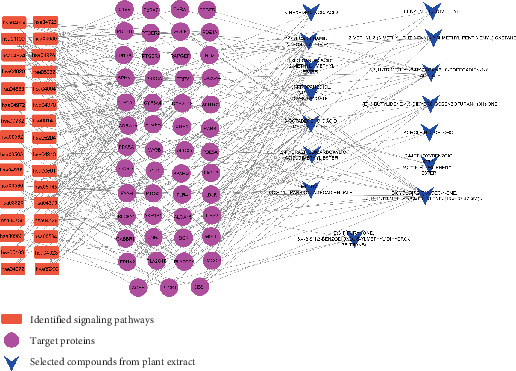
Overview of STC networks. Orange square: identified signaling pathways; pink circle: target proteins; and blue triangle: selected compounds from plant extract.

Based on the comprehensive analysis of the data, an STC network was constructed, integrating the information from 32 signaling pathways, 51 target proteins, and 15 compounds (Figure 6) [45]. In this network, the nodes represent three key elements: signaling pathways, target proteins, and compounds. The STC network analysis provided valuable insights into the therapeutic potential of these compounds against AD, as supported by the interaction of the principal target proteins (PTGS2, CYP2C19, and PLA2G4A) associated with AD. Notably, the STC network analysis identified five key compounds based on their degree of interaction with both compounds and target proteins. These key compounds are as follows: (E)-3-butylidene-4,5-dihydroisobenzofuran-1(3H)-one, bicyclo[7.2.0]undec-4-ene, 4,11,11-trimethyl-8-methylene-,[1R-(1R∗,4Z,9S∗)]-, 2-methoxybenzoic acid, 2,3-dichlorophenyl ester, 1,3-propanediol, 2-methyl-, dipropanoate, and 1,2-hydrazinedicarboxylic acid, dimethyl ester. These compounds emerged as key candidates based on their significant interactions within the STC network, highlighting their potential therapeutic relevance in the context of AD.

### 3.7. Molecular Docking of Three Target Proteins and Five Compounds Involved in the Metabolic Pathway

**Figure 7:**
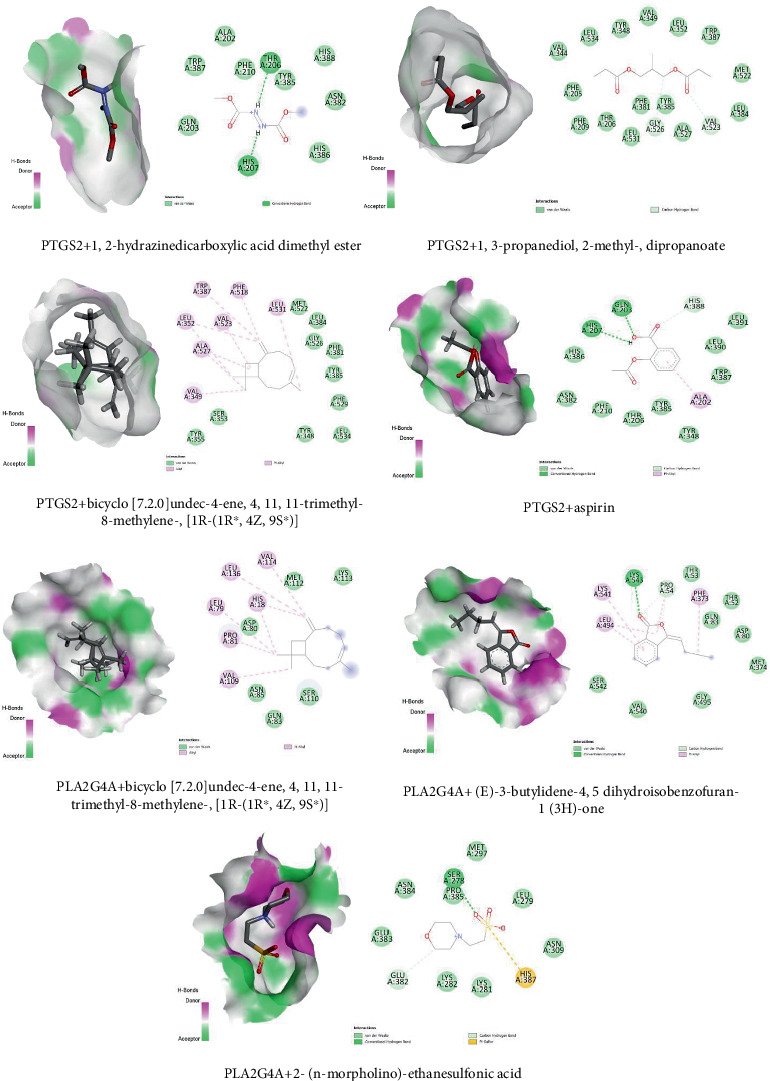
Interactions among selected compounds and control drugs with targeted PTGS2 and PLA2G4A protein.

A molecular docking test (MDT) was conducted to assess the binding interactions between the PTGS2 protein (PDB ID: 5F19) and three compounds associated with the metabolic signaling pathway. Among these compounds, bicyclo[7.2.0]undec-4-ene, 4,11,11-trimethyl-8-methylene-,[1R-(1R∗,4Z,9S∗)], displayed the highest binding energy with the PTGS2 protein (PDB ID: 5F19) at −8 kcal/mol, followed by 1,2-hydrazinedicarboxylic acid, dimethyl ester at −5 kcal/mol and 1,3-propanediol, 2-methyl-, dipropanoate at −6 kcal/mol. Notably, bicyclo[7.2.0]undec-4-ene, 4,11,11-trimethyl-8-methylene-,[1R-(1R∗,4Z,9S∗)] exhibited a greater affinity compared to the positive control (aspirin) with a binding energy of −6.4 kcal/mol. The docking details of these three compounds and the positive control are presented in Figure 7 and Table 6.

Furthermore, the MDT analysis of two compounds, (E)-3-butylidene-4,5-dihydroisobenzofuran-1(3H)-one and bicyclo[7.2.0]undec-4-ene, 4,11,11-trimethyl-8-methylene-,[1R-(1R∗,4Z,9S∗)], was conducted on the PLA2G4A protein (PDB ID: 1CJY) to determine their binding affinity. Both compounds exhibited higher binding affinities (−5.0 kcal/mol) compared to the positive control, 2-(*n*-morpholino)ethanesulfonic acid, which had a binding energy of −4.3 kcal/mol (Figure 7 and Table 6).

Lastly, the MDT analysis of a CYP2C19 protein (PDB ID: 4GQS) was performed to assess the affinity of one compound. The compound, 2-methoxybenzoic acid, 2,3-dichlorophenyl ester, demonstrated the highest binding energy of −7 kcal/mol when docked with the CYP2C19 protein (PDB ID: 4GQS). However, this binding energy was lower than the positive control, (2-methyl-1-benzofuran-3-yl)-(4-hydroxy-3,5-dimethylphenyl)methanone, which exhibited a binding energy of −8.6 kcal/mol.

Docking analysis showed that 1,2-hydrazinedicarboxylic acid dimethyl ester formed conventional hydrogen bonds with HIS207 and THR206 in PTGS2. 1,3-Propanediol, 2-methyl-, dipropanoate engaged in carbon–hydrogen bonding with VAL523 and GLY526. Bicyclo[7.2.0]undec-4-ene, 4,11,11-trimethyl-8-methylene- formed Pi-alkyl interactions with TRP387 and PHE518. Aspirin formed conventional hydrogen bonds with GLN203 and HIS207 (Table 6).

For PLA2G4A, bicyclo[7.2.0]undec-4-ene, 4,11,11-trimethyl-8-methylene- exhibited alkyl and Pi-alkyl interactions with VAL114, LEU136, PRO81, and HIS18. 3-Butylidene-4,5-dihydroisobenzofuran-1(3H)-one showed a conventional hydrogen bond with LYS543 and Pi-alkyl interactions with PHE373. 2-(*N*-Morpholino)ethanesulfonic acid formed a conventional hydrogen bond with SER278 and a carbon–hydrogen bond with GLU382 (Table 6).

In the CYP2C19 complex, 2-methoxybenzoic acid, 2,3-dichlorophenyl ester showed carbon–hydrogen bonding with ASN107 and Pi-alkyl interactions with ALA103, LEU102, and PHE114. (4-Hydroxy-3,5-dimethylphenyl)(2-methyl-1-benzofuran-3-yl)methanone displayed carbon–hydrogen bonding with PHE428, Pi-sulfur bonding with CYS435, and Pi-alkyl interactions with PHE476, LEU366, and ILE362 (Table 6).

### 3.8. Molecular Dynamics (MD) Simulation

**Figure 8:**
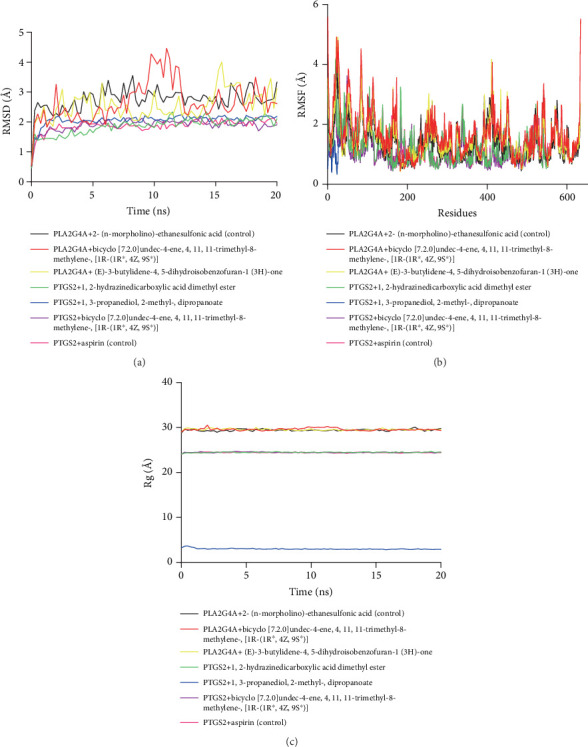
Molecular dynamics (MD) simulation of proteins with chemical complexes. (a) RMSD. (b) RMSF. (c) Rg.

To assess the structural variation of the docked complexes, MD simulation trajectories were analyzed to evaluate the RMSD, RMSF, and Rg (Figure 8). The RMSD profiles of the PTGS2 protein complexes, including the positive control (aspirin), exhibited minimal deviations with a consistently straight trajectory. However, the RMSD profile of the PLA2G4A protein complexes showed an elevated level of fluctuations, indicating a higher degree of flexibility among the complexes. Specifically, bicyclo[7.2.0]undec-4-ene, 4,11,11-trimethyl-8-methylene-,[1R-(1R∗,4Z,9S∗)] exhibited slightly higher deviations around the 10 ns simulation period, although these fluctuations did not exceed 3.0 Å in the RMSD analysis.

The RMSF values for most amino acid residues in the docked complexes were below 3 Å, indicating a relatively lower level of flexibility in the protein–phytochemical complexes. Notably, the PTGS2 + 1,3-propanediol, 2-methyl-, dipropanoate complex exhibited the lowest RMSF profile. In contrast, the PLA2G4A complexes displayed a higher RMSF profile, suggesting looser packaging systems for the molecules. These findings indicate that the PTGS2 complexes are more tightly packed compared to the PLA2G4A complexes.

**Figure 9:**
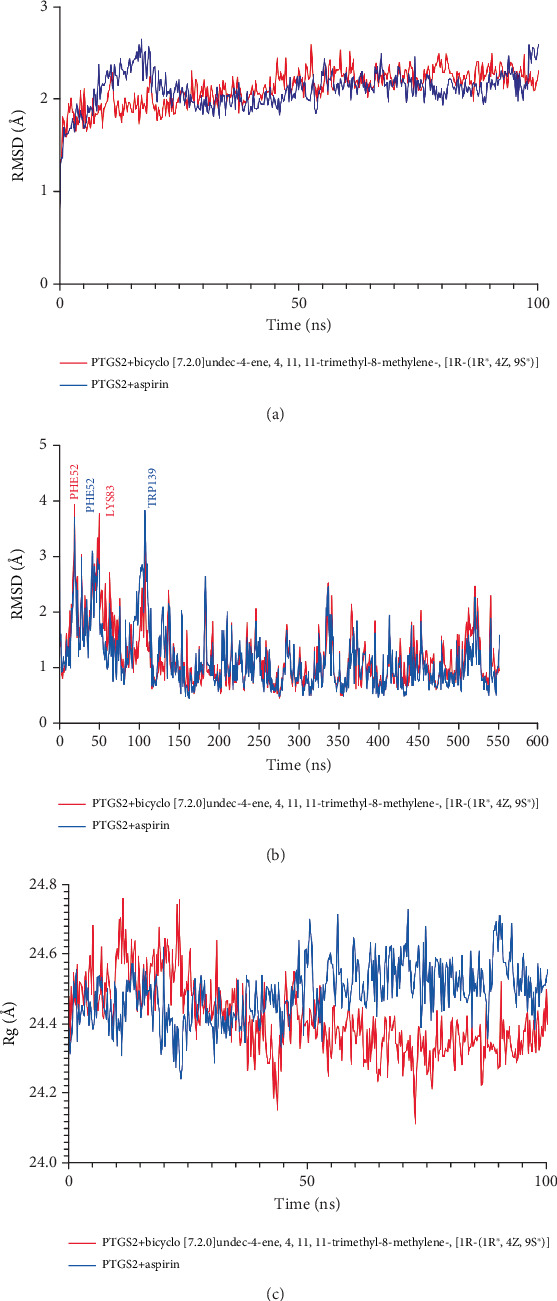
Molecular dynamics simulation of the protein–ligand complex for 100 ns. (a) RMSD. (b) RMSF. (c) Rg.

From the 20 ns simulation, it was found that the interaction between PTGS2 and bicyclo[7.2.0]undec-4-ene, 4,11,11-trimethyl-8-methylene-,[1R-(1R∗,4Z,9S∗)] showed a more stable interaction compared to other compounds. Based on this result, the PTGS2 + bicyclo[7.2.0]undec-4-ene, 4,11,11-trimethyl-8-methylene-,[1R-(1R∗,4Z,9S∗)] complex and its control PTGS2 + aspirin complex were extended for a 100 ns simulation. The PTGS2 + bicyclo[7.2.0]undec-4-ene, 4,11,11-trimethyl-8-methylene-,[1R-(1R∗,4Z,9S∗)] complex exhibited relatively stable RMSD values throughout the 100 ns simulation, with minor fluctuations observed around 2–4, 25–30, and 65–80 ns (Figure 9).

In contrast, the PTGS2 + aspirin complex showed greater fluctuations after 40 ns, with notable instability peaks between 45 and 60 ns and around 90 and 100 ns, indicating higher conformational changes compared to the bicyclo[7.2.0]undec-4-ene, 4,11,11-trimethyl-8-methylene-,[1R-(1R∗,4Z,9S∗)] complex. The Rg profiles for both complexes showed slight fluctuation in compactness between 50–60 and 70–80 ns. In the PTGS2 + aspirin complex, residues PHE52, PHE74, LEU75, TRP139, and GLU140 showed RMSF values exceeding 3 Å, indicating higher flexibility in these regions. For the PTGS2 + bicyclo[7.2.0]undec-4-ene, 4,11,11-trimethyl-8-methylene-,[1R-(1R∗,4Z,9S∗)] complex, only residues PHE52, PHE81, LYS83, and TRP139 exhibited fluctuations crossing 3 Å, suggesting comparatively reduced flexibility compared to the control (Figure 9).

### 3.9. Quantum Chemistry of Key Ingredients

**Figure 10:**
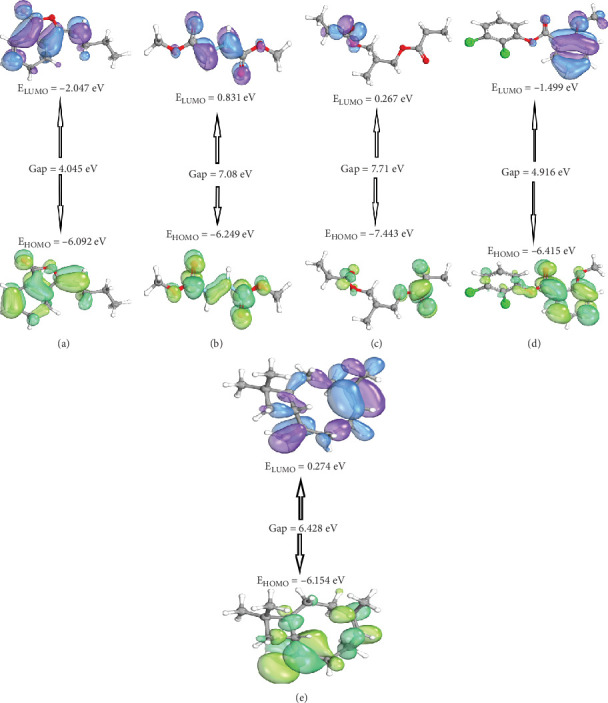
The structure of the docked compounds with their HOMO and LUMO energy. (a) (E)-3-Butylidene-4,5-dihydroisobenzofuran-1(3H)-one. (b) 1,2-Hydrazinedicarboxylic acid, dimethyl ester. (c) 1,3-Propanediol, 2-methyl-, dipropanoate. (d) 2-Methoxybenzoic acid, 2,3-dichlorophenyl ester. (e) Bicyclo[7.2.0]undec-4-ene, 4,11,11-trimethyl-8-methylene-,[1R-(1R∗,4Z,9S∗)].

The electron-donating and accepting properties of molecules can be characterized by their HOMO and LUMO, respectively. In this study, the energies of the HOMO, LUMO, and their energy gaps were calculated for the compounds under investigation. Among the molecules studied, 1,3-propanediol, 2-methyl-, dipropanoate exhibited the highest energy level for its HOMO, indicating a relatively higher electron-donating tendency. On the other hand, (E)-3-butylidene-4,5-dihydroisobenzofuran-1(3H)-one displayed the lowest energy for its HOMO, suggesting a greater electron-accepting ability (Figure 10). In simpler terms, (E)-3-butylidene-4,5-dihydroisobenzofuran-1(3H)-one exhibited higher reactivity compared to 1,3-propanediol, 2-methyl-, dipropanoate, which showed lower reactivity. The energy gaps between HOMO and LUMO further support these observations, indicating that the compounds' reactivity is consistent with their respective electron-donating and accepting tendencies (Table 7).

## 4. Discussion

Nature-based treatment options, particularly plant materials, are highly encouraged for the treatment of various diseases, including AD, due to their minimal side effects and high efficacy. Analyzing the drug likeness of plant-derived compounds specific to diseases is a prerequisite in this regard. Hence, the present study is aimed at contributing to global AD-related treatment research by screening potential drug leads and revealing the underlying mechanisms of action for the development of alternative effective treatments.

*P. chaba*, a traditional culinary spice widely used in Bangladesh, particularly in the southern region, has been reported to possess numerous medicinal properties, making it a promising candidate for AD treatment. Previous studies have hypothesized the multifactorial nature of AD pathogenesis, suggesting that the “one target, one drug” approach may be insufficient for effectively treating this complex disease [46, 47]. Additionally, the presence of numerous chemical components in plants makes it challenging to assess the effectiveness of herbal treatments using the traditional “one drug, one target” method [5]. Therefore, employing network pharmacology studies to design treatments for complex diseases, including AD, is beneficial in leveraging the potential of medicinal plants [47, 48].

In this study, 15 compounds were identified from the GC-MS analysis of *P. chaba* based on their ADME properties and other adopted bioinformatics approaches. Subsequently, PPI network analysis was performed to identify key target proteins and their interactions with different signaling pathways in AD. PTGS2, CYP2C19, and PLA2G4A were identified as prominent target proteins with potential therapeutic implications for AD. The involvement of these proteins in AD progression has been reported in several studies [49–51]. PTGS2 is linked to neuroinflammation, as its upregulation near amyloid plaques promotes oxidative stress and neuronal damage [51]. PLA2G4A plays a role in lipid metabolism and inflammatory signaling, contributing to synaptic dysfunction in AD [50]. CYP2C19 is involved in drug metabolism and A*β* processing, with polymorphisms affecting disease progression [49].

Furthermore, STC network analysis was employed to investigate the association of these target proteins with the metabolic signaling pathway. The metabolic pathway, with a rich factor of 0.024, was found to be strongly associated with PTGS2, PLA2G4A, and CYP2C19 proteins. Interestingly, PTGS2 was not only found to be involved in the metabolic pathway but also demonstrated an association with cancer development [52]. Based on the STC network analysis, PTGS2 emerged as the primary target, as it was enriched in 13 out of 32 signaling pathways and exhibited interactions with some of the prominent compounds from *P. chaba*. The inactivation of PTGS2 is considered a potential mechanistic approach to counter neural degeneration and excitotoxicity in AD pathogenesis [53]. Similarly, effective blocking of PLA2G4A activation, which impairs autophagy flux by reducing lysosomal damage, could contribute to preventing neural cell death [54]. Although the involvement of CYP2C19 proteins in AD pathophysiology is relatively less explored, they could serve as potential targets due to their role in amyloid metabolism [55]. Moreover, the STC network analysis identified five compounds from the initial screening of 15 compounds from the plant extract that exhibited strong interactions with the metabolic pathway and the three target proteins.

MDT was performed between the three target proteins and the five potential compounds to validate their efficacy. The MDT values were compared with positive controls for each target protein. The MDT revealed that several phytocompounds exhibited favorable binding affinities with key inflammatory and metabolic proteins, indicating potential therapeutic relevance. The docking results revealed that bicyclo[7.2.0]undec-4-ene showed the strongest binding to PTGS2 (5F19) with a binding energy of −8.0 kcal/mol, outperforming the standard drug aspirin (−6.4 kcal/mol). Similarly, both bicyclo[7.2.0]undec-4-ene and (E)-3-butylidene-4,5-dihydroisobenzofuran displayed better binding to PLA2G4A (1CJY) than the control compound, indicating promising interaction with this target as well. Previous studies suggest that compounds with more negative binding energies tend to form more stable and effective interactions with their targets [56, 57]. These findings highlight the potential of the tested compounds as promising candidates for further biological validation.

Among the tested compounds, bicyclo[7.2.0]undec-4-ene, 4,11,11-trimethyl-8-methylene- consistently showed strong interactions with both PTGS2 and PLA2G4A, surpassing the binding affinity of standard controls like aspirin and 2-(*N*-morpholino)ethanesulfonic acid. This suggests a possible multitarget interaction profile. The strength of these docking complexes is supported not only by their binding energy values but also by the presence of specific noncovalent interactions, such as Pi-alkyl and hydrophobic contacts, which are crucial for ligand stabilization within the binding pocket [58]. Similarly, 2-methoxybenzoic acid, 2,3-dichlorophenyl exhibited notable binding with CYP2C19, although its affinity was slightly lower than that of the positive control. Hydrogen bonds, carbon–hydrogen interactions, and Pi-alkyl contacts observed in various complexes further validate their structural compatibility with the active site residues [59]. These interactions are essential because they contribute significantly to binding specificity and overall complex stability, often determining the biological activity of a compound [60]. These findings highlight key candidates that warrant further in vitro and in vivo exploration to validate their anti-inflammatory or metabolic regulatory potential.

MD simulation analysis showed that the PTGS2 complexes maintained consistently stable RMSD profiles, suggesting good structural integrity throughout the simulation [61]. In contrast, the PLA2G4A complexes showed more fluctuations, indicating increased flexibility [62]. Notably, the bicyclo[7.2.0]undec-4-ene–PLA2G4A complex had a slight RMSD peak around 10 ns but remained within a stable range (< 3.0 Å). RMSF analysis further supported this, showing lower residue fluctuations in PTGS2 complexes, especially with 1,3-propanediol, 2-methyl-, dipropanoate compared to the more flexible PLA2G4A complexes. These results suggest that PTGS2 complexes are more tightly packed and structurally stable, which is supported by their lower RMSD and RMSF values, pointing to stronger and potentially more effective drug–target interactions [61, 62].

The MD analysis of 100 ns indicated that bicyclo[7.2.0]undec-4-ene, 4,11,11-trimethyl-8-methylene-,[1R-(1R∗,4Z,9S∗)] formed a more stable complex with PTGS2 compared to aspirin. RMSD and Rg profiles demonstrated lower fluctuations in the bicyclo[7.2.0]undec-4-ene, 4,11,11-trimethyl-8-methylene-,[1R-(1R∗,4Z,9S∗)] complex throughout the 100 ns simulation. Fewer residues showed high flexibility (RMSF > 3 Å) in the bicyclo[7.2.0]undec-4-ene, 4,11,11-trimethyl-8-methylene-,[1R-(1R∗,4Z,9S∗)] complex, suggesting enhanced structural stability [63, 64]. These findings support its potential as a promising candidate for PTGS2 targeting [61].

Among the tested compounds, (E)-3-butylidene-4,5-dihydroisobenzofuran-1(3H)-one demonstrated the lowest HOMO-LUMO gap (4.045 eV), suggesting higher chemical flexibility, which is often advantageous for adaptive binding with protein targets [65]. Interestingly, bicyclo[7.2.0]undec-4-ene, 4,11,11-trimethyl-8-methylene- showed a moderately low gap (6.428 eV) alongside a favorable softness value (0.3111 eV), indicating a balanced degree of chemical reactivity and stability. This profile supports its ability to interact effectively with multiple target proteins while maintaining structural integrity [58, 65]. In contrast, more rigid compounds like 1,3-propanediol, 2-methyl-, dipropanoate and 1,2-hydrazinedicarboxylic acid, dimethyl ester exhibited higher energy gaps, suggesting limited adaptability. The chemical profile of bicyclo[7.2.0]undec-4-ene aligns well with its strong docking performance and stable MD simulation behavior, reinforcing its potential as a promising multitarget lead compound in AD drug discovery [66].

The functional feasibility and atomic-level interactions were supported by MDS and quantum chemistry analysis, which involved the interpretation of RMSD and least energy conformation, respectively [67]. Among the five compounds, bicyclo[7.2.0]undec-4-ene, 4,11,11-trimethyl-8-methylene-,[1R-(1R∗,4Z,9S∗)] exhibited significant binding efficacy with PTGS2 and PLA2G4A, displaying higher binding energy compared to their respective controls, indicating their potential in AD treatment. This compound, commonly known as caryophyllene, is a naturally occurring sesquiterpene widely recognized for its diverse biological activities and industrial applications [68–70]. It has been isolated from various plants and is reported to exhibit both antimicrobial and anti-inflammatory properties [69]. On the other hand, 2-methoxybenzoic acid, 2,3-dichlorophenyl ester exhibited slightly lower binding energy compared to the control. However, the lower binding energy of this compound against CYP2C19 could be overlooked, considering the potential adverse effects of synthetic drugs [71]. Several plant-derived bioactive compounds have demonstrated efficacy in halting neurodegeneration through diverse mechanisms, and some of them hold promise as potential drug leads for AD [72, 73].

Therefore, modulation of key target proteins (PTGS2, PLA2G4A, and CYP2C19) within the metabolic signaling pathway, which plays roles in AD pathophysiology, using compounds identified from the *P. chaba* plant extract, could enhance the current treatment options for AD. This research provides clear direction for researchers to investigate the implementation of these compounds in animal models for neurodegenerative diseases, particularly AD, to develop safe and effective treatments. While the computational findings provide promising insights into the potential interaction of the identified compounds with AD-related targets, further validation is essential. Future in vitro and in vivo clinical studies are required to confirm the bioactivity of these compounds at the cellular level. These steps are critical to translating the current in silico results into viable therapeutic strategies.

## 5. Conclusion

The present study employed a pharmacological network approach to investigate the potential of *P. chaba* in countering AD. Through an analysis of various signaling pathways, target proteins, and compounds, significant findings were obtained. The involvement of a key signaling pathway, namely, the metabolic signaling pathway, along with three important target proteins (PTGS2, CYP2C19, and PLA2G4A) and five identified compounds, was identified. Comparative analysis with positive controls revealed that the three compounds from *P. chaba* exhibited higher affinity towards their respective target proteins, suggesting their potential as novel anti-Alzheimer's agents. Furthermore, the quantum chemistry analysis indicated their superior chemical reactivity compared to conventional medications. Consequently, our research strategy holds promises for advancing network pharmacology investigations on herbal plants for the treatment of AD.

## Figures and Tables

**Table 1:** The target protein site's center and dimensions.

**Protein name**	**PDB ID**	**Grid box**		
		**X**	**Y**	**Z**
PTGS2	5F19	16.9276	41.8	58.3518
CYP2C19	4GQS	−70.81	21.0071	−46.156
PLA2G4A	1CJY	72.7126	25.0514	59.6409

**Table 2:** Phytochemicals identified from *P. chaba* via GC-MS and profiling of biological activities.

**No.**	**Compounds**	**PubChem ID**	**Retention time (min)**	**Area (%)**	**Molecular formula**
1	1,3-Propanediol, 2-methyl-, dipropanoate	345905	3.342	5.91287e−07	C_10_H_18_O_4_
2	Benzene, 1,3-dimethyl-	7929	4.092	7.23981e−07	C_6_H_4_(CH_3_)_2_
3	Acrolein, 2-chloro-	560790	6.143	1.08686e−06	C_3_H_3_ClO
4	1,2-Hydrazinedicarboxylic acid, dimethyl ester	265934	8.462	1.49715e−06	C_4_H_8_NO_4_
5	Bicyclo[7.2.0]undec-4-ene, 4,11,11-trimethyl-8-methylene-,[1R-(1R∗,4Z,9S∗)]-	5322111	16.378	2.89769e−06	C_15_H_24_
6	3,7,11-Trimethyl-3-hydroxy-6,10-dodecadien-1-yl acetate	5366047	20.158	3.57001e−06	C_15_H_30_O_3_
7	2-Methyl-3-(3-methyl-but-2-enyl)-2-(4-methyl-pent-3-enyl)-oxetane	550119	21.592	3.82019e−06	C_15_H_26_O
8	(E)-3-Butylidene-4,5-dihydroisobenzofuran-1(3H)-one	5877292	26.793	4.74038e−06	C_12_H_14_O_2_
9	Tridecanoic acid, 12-methyl-, methyl ester	21204	27.289	4.82813e−06	C_15_H_30_O_2_
10	*N*-Hexadecanoic acid	985	28.301	5.00718e−06	C_16_H_32_O_2_
11	2,4-Decadienamide, *N*-isobutyl-, (E,E)-	5318516	29.52	5.22286e−06	C_14_H_25_NO
12	13-Octadecenoic acid, methyl ester	5364506	30.873	5.46224e−06	C_19_H_36_O_2_
13	Methyl 9-cis,11-trans-octadecadienoate	11548436	31.088	5.50028e−06	C_19_H_34_O_2_
14	2(3H)-Furanone, 3,4-bis(1,3-benzodioxol-5-ylmethyl)dihydro-, (3R-trans)-	11002708	33.319	5.895e−06	C_20_H_18_O_6_
15	2-Methoxybenzoic acid, 2,3-dichlorophenyl ester	91514954	33.601	5.95189e−06	C_14_H_10_C_l2_O_3_

**Table 3:** Physicochemical characteristics of the compounds that promote oral bioavailability and permeability of the cell membrane.

**Compounds**	**Lipinski's rule**				**Lipinski's violation**	**Bioavailability score**	**TPSA (Å** ^**2**^ **)**
	**Molecular weight**	**Hydrogen bond acceptor**	**Hydrogen bond donor**	**M Logp**			
Standard	< 500	< 10	≤ 5	≤ 4.15	≤ 1	> 0.1	< 140
1,3-Propanediol, 2-methyl-, dipropanoate	202.25	4	0	1.55	0	0.55	52.60
Benzene, 1,3-dimethyl-	106.15	0	0	3.85	0	0.55	0
Acrolein, 2-chloro-	90.51	1	0		0	0.49	15.07
1,2-Hydrazinedicarboxylic acid, dimethyl ester	148.12	4	2	−0.56	0	0.55	76.66
Bicyclo[7.2.0]undec-4-ene, 4,11,11-trimethyl-8-methylene-,[1R-(1R∗,4Z,9S∗)]-	204.35	0	0	4.63	1	0.55	0
3,7,11-Trimethyl-3-hydroxy-6,10-dodecadien-1-yl acetate	282.42	3	1	3.35	0	0.55	46.53
2-Methyl-3-(3-methyl-but-2-enyl)-2-(4-methyl-pent-3-enyl)-oxetane	222.37	1	0	3.56	0	0.55	9.23
(E)-3-Butylidene-4,5-dihydroisobenzofuran-1(3H)-one	190.24	2	0	2.47	0	0.55	26.3
Tridecanoic acid, 12-methyl-, methyl ester	242.40	2	0	3.94	0	0.55	26.3
*n*-Hexadecanoic acid	256.42	2	1	4.19	1	0.85	37.3
2,4-Decadienamide, *n*-isobutyl-, (E,E)-	223.35	1	1	3.08	0	0.55	29.1
13-Octadecenoic acid, methyl ester	296.49	2	0	4.7	1	0.55	26.3
Methyl 9-cis,11-trans-octadecadienoate	294.47	2	0	4.8	1	0.55	26.3
2(3H)-Furanone, 3,4-bis(1,3-benzodioxol-5-ylmethyl)dihydro-, (3R-trans)-	354.35	6	0	2.71	0	0.55	63.22
2-Methoxybenzoic acid, 2,3-dichlorophenyl ester	297.13	3	0	4.11	0	0.55	35.53

**Table 4:** The degree values of 60 targets.

**Serial**	**Gene symbol**	**Degree**
1	PTGS2	22
2	PPARA	19
3	PPARG	18
4	PLA2G4A	16
5	CYP2C19	15
6	CNR1	13
7	CYP3A4	13
8	FAAH	12
9	PLA2G1B	12
10	PTGS1	12
11	TRPV1	12
12	ALOX15	11
13	ALOX5	11
14	MGLL	11
15	TLR4	11
16	CYP1A2	10
15	PLA2G2A	10
18	ACHE	6
19	CES1	6
20	EPHX2	6
21	LDLR	6
22	MAOB	6
23	PTGER3	6
24	SLC6A4	6
25	CNR2	5
26	GSR	5
27	HMGCR	5
28	NAAA	5
29	PRKCA	5
30	RORA	0
31	CTSB	4
32	FABP3	4
33	PDE5A	4
34	PTGER2	4
35	RAPGEF4	4
36	SPHK1	4
37	CES2	3
38	ENPP2	3
39	GABBR1	3
40	P2RX7	3
41	PDE4A	3
42	PPARD	3
43	PTGFR	3
51	SLC6A2	3
45	ADRA1A	2
46	EDNRA	2
47	PDE4D	2
48	SQLE	2
49	CDC25B	1
50	GLI1	1
51	GLI2	1
52	GSTK1	1
53	RARB	1
54	RGS4	1
55	THRA	1
56	AKR1C2	0
57	KDM4A	0
58	POLB	0
59	PTPN13	0
60	SHBG	0

Abbreviations: ACHE, acetylcholinesterase; activated, receptor delta; acylethanolamine, acid amidase; ADR, A; adrenoceptor, alpha; AK, R; ald, o; ALO, X; also, known as cyclooxygenase; aminobutyric, acid Type B receptor Subunit; arachidonat, e; binding, globulin; cannabinoid, Receptor; Carboxylesteras, e; CD, C; CE, S; cell, division cycle; CN, R; CoA, reductase; CTSB, cathepsin B; CY, P; cytochrome, P; cytosolic, calcium dependent; density, lipoprotein receptor; disulfide, reductase; ectonucleotide, pyrophosphatase; EDNRA, endothelin receptor Type A; endoperoxide, Synthase; ENP, P; EPH, X; Epoxide, Hydrolase; FAAH, fatty acid amide hydrolase; FAB, P; Famil, y; fatty, acid; GABB, R; gamm, a; GL, I; GLI, family zinc Finger; glutathione, S; Group, IIA; GSR, glutathione; GST, K; HMGC, R; hydrox, y; KD, M; keto, reductase Family; LDLR, low; like, Receptor; lipoxygenas, e; lysine, demethylase; MAOB, Monoamine Oxidase B; Membe, r; Member, C; methylglutary, l; MGLL, monoglyceride lipase; NAAA, N; nonreceptor, Type; PD, E; phosphodiesteras, e; Phosphodiesteras, e; phospholipase, A; PL, A; POLB, DNA polymerase beta; PPARA, peroxisome proliferator; PPARD, peroxisome proliferator; PPARG, peroxisome proliferator; PRKCA, protein kinase C alpha; prostaglandi, n; prostaglandin, E Receptor; protein, tyrosine phosphatase; PTG, S; PTGE, R; PTGFR, prostaglandin F receptor; PTP, N; purinergic, receptor P; Rap, guanine nucleotide exchange Factor; RAPGE, F; RARB, retinoic acid receptor beta; regulator, of G; related, orphan receptor A; RG, S; RORA, RAR; SHBG, sex hormone; SL, C; solute, carrier Family; SPH, K; sphingosine, Kinase; SQLE, squalene epoxidase; Subfamily, A Member; THRA, thyroid hormone receptor alpha; TL, R; tol, l; transferase, Kappa; transient, receptor potential Vanilloid; TRP, V.

**Table 5:** Target proteins and signaling pathways enrichment associated with AD.

**KEGG ID**	**False discovery rate**	**Proteins**
hsa00590	2.36e−10	PLA2G1B, PTGS1, PLA2G4A, PTGS2, CYP2C19, ALOX5, PLA2G2A, EPHX2, ALOX15
hsa00591	2.27e−09	PLA2G1B, CYP3A4, CYP1A2, PLA2G4A, CYP2C19, PLA2G2A, ALOX15
hsa04726	8.93e−09	SLC6A4, PTGS1, PLA2G4A, PTGS2, CYP2C19, ALOX5, MAOB, PRKCA, ALOX15
hsa01100	5.00e−07	GSR, MGLL, SQLE, HMGCR, PLA2G1B, SPHK1, CYP3A4, CYP1A2, PDE4D, PDE5A, PTGS1, PLA2G4A, PTGS2, CYP2C19, ALOX5, MAOB, PDE4A, PLA2G2A, EPHX2, ALOX15
hsa04080	5.00e−07	PTGER2, THRA, EDNRA, P2RX7, PTGER3, CNR1, PTGFR, CNR2, GABBR1, ADRA1A, TRPV1
hsa04024	1.06e−06	GLI1, PTGER2, EDNRA, PDE4D, PTGER3, GABBR1, PDE4A, RAPGEF4, PPARA
hsa04020	0.00012	SPHK1, EDNRA, P2RX7, PTGER3, PTGFR, ADRA1A, PRKCA
hsa04270	0.00015	PLA2G1B, EDNRA, PLA2G4A, ADRA1A, PLA2G2A, PRKCA
hsa05200	0.00015	GLI1, PTGER2, PPARG, PPARD, EDNRA, RARB, PTGER3, PTGS2, GLI2, PRKCA
hsa05204	0.00015	CYP3A4, CYP1A2, PTGS2, CYP2C19, AKR1C2
hsa04913	0.00068	PLA2G4A, PTGS2, ALOX5, LDLR
hsa04923	0.00083	MGLL, PTGER3, PTGS1, PTGS2
hsa04370	0.00093	SPHK1, PLA2G4A, PTGS2, PRKCA
hsa00982	0.0013	CYP3A4, CYP1A2, CYP2C19, MAOB
hsa00592	0.0019	PLA2G1B, PLA2G4A, PLA2G2A
hsa03320	0.002	PPARG, PPARD, FABP3, PPARA
hsa04072	0.002	SPHK1, PLA2G4A, PTGFR, RAPGEF4, PRKCA
hsa04723	0.002	FAAH, MGLL, PTGS2, CNR1, PRKCA
hsa05032	0.0033	PDE4D, GABBR1, PDE4A, PRKCA
hsa04750	0.0039	PTGER2, PLA2G4A, PRKCA, TRPV1
hsa00564	0.0041	ACHE, PLA2G1B, PLA2G4A, PLA2G2A
hsa00565	0.0073	PLA2G1B, PLA2G4A, PLA2G2A
hsa00140	0.0132	CYP3A4, CYP1A2, AKR1C2
hsa04664	0.0153	PLA2G4A, ALOX5, PRKCA
hsa04924	0.0153	PTGER2, EDNRA, CTSB
hsa00983	0.0227	CES2, CYP3A4, CES1
hsa04666	0.035	SPHK1, PLA2G4A, PRKCA
hsa04976	0.035	HMGCR, CYP3A4, LDLR
hsa04972	0.0424	PLA2G1B, PLA2G2A, PRKCA
hsa04928	0.0469	PDE4D, PDE4A, PRKCA
hsa05145	0.0479	TLR4, ALOX5, LDLR
hsa05163	0.0497	PTGER2, PTGER3, PTGS2, PRKCA

**Table 6:** Target compounds and positive controls with binding energy.

**Protein**	**Ligand**	**PubChem ID**	**Binding energy**	**Interacting residues**	**Type**	**Type of bond**
PTGS2 (PDB ID: 5F19)	1,2-Hydrazinedicarboxylic acid dimethyl ester	265934	-5.0 kcal/mol	ALA202	Hydrophobic interactions	Van der Waals
				TRP387	Hydrophobic interactions	Van der Waals
				GLN203	Hydrophobic interactions	Van der Waals
				HIS207	Hydrogen bond	Conventional hydrogen bond
				HIS386	Hydrophobic interactions	Van der Waals
				ASN382	Hydrophobic interactions	Van der Waals
				HIS388	Hydrophobic interactions	Van der Waals
				TYR385	Hydrophobic interactions	Van der Waals
				THR206	Hydrogen bond	Conventional hydrogen bond
				PHE210	Hydrophobic interactions	Van der Waals
	1,3-Propanediol, 2-methyl-, dipropanoate	345905	-6.0 kcal/mol	VAL344	Hydrophobic interactions	Van der Waals
				LEU534	Hydrophobic interactions	Van der Waals
				TYR348	Hydrophobic interactions	Van der Waals
				VAL349	Hydrophobic interactions	Van der Waals
				LEU352	Hydrophobic interactions	Van der Waals
				TRP387	Hydrophobic interactions	Van der Waals
				MET522	Hydrophobic interactions	Van der Waals
				LEU384	Hydrophobic interactions	Van der Waals
				VAL523	Hydrogen bond	Carbon–hydrogen bond
				ALA527	Hydrophobic interactions	Van der Waals
				TYR385	Hydrophobic interactions	Van der Waals
				GLY526	Hydrogen bond	Carbon–hydrogen bond
				PHE381	Hydrophobic interactions	Van der Waals
				LEU531	Hydrophobic interactions	Van der Waals
				THR206	Hydrophobic interactions	Van der Waals
				PHE209	Hydrophobic interactions	Van der Waals
				PHE205	Hydrophobic interactions	Van der Waals
	Bicyclo[7.2.0]undec-4-ene, 4,11,11-trimethyl-8-methylene-,[1R-(1R∗,4Z,9S∗)]	5322111	-8.0 kcal/mol	TRP387	Hydrophobic interactions	Pi-alkyl
				PHE518	Hydrophobic interactions	Pi-alkyl
				LEU531	Hydrophobic interactions	Alkyl
				VAL523	Hydrophobic interactions	Alkyl
				LEU352	Hydrophobic interactions	Alkyl
				ALA527	Hydrophobic interactions	Alkyl
				VAL349	Hydrophobic interactions	Pi-alkyl
				SER353	Hydrophobic interactions	Van der Waals
				TYR355	Hydrophobic interactions	Van der Waals
				TYR348	Hydrophobic interactions	Van der Waals
				LEU534	Hydrophobic interactions	Van der Waals
				PHE529	Hydrophobic interactions	Van der Waals
				TYR385	Hydrophobic interactions	Van der Waals
				PHE381	Hydrophobic interactions	Van der Waals
				GLY526	Hydrophobic interactions	Van der Waals
				LEU384	Hydrophobic interactions	Van der Waals
				MET522	Hydrophobic interactions	Van der Waals
	Aspirin	2251	-6.4 kcal/mol	GLN203	Hydrogen bond	Conventional hydrogen bond
				HIS207	Hydrogen bond	Conventional hydrogen bond
				HIS386	Hydrophobic interactions	Van der Waals
				ASN382	Hydrophobic interactions	Van der Waals
				PHE210	Hydrophobic interactions	Van der Waals
				THR206	Hydrophobic interactions	Van der Waals
				TYR385	Hydrophobic interactions	Van der Waals
				TYR348	Hydrophobic interactions	Van der Waals
				ALA202	Hydrophobic interactions	Pi-alkyl
				TRP387	Hydrophobic interactions	Van der Waals
				LEU390	Hydrophobic interactions	Van der Waals
				LEU391	Hydrophobic interactions	Van der Waals
				HIS388	Hydrogen bond	Carbon–hydrogen bond

PLA2G4A (PDB ID: 1CJY)	Bicyclo[7.2.0]undec-4-ene, 4,11,11-trimethyl-8methylene-,[1R-(1R∗,4Z,9S∗)]	5322111	-5.0 kcal/mol	VAL114	Hydrophobic interactions	Alkyl
				LEU136	Hydrophobic interactions	Alkyl
				HIS18	Hydrophobic interactions	Pi-alkyl
				LEU79	Hydrophobic interactions	Alkyl
				PRO81	Hydrophobic interactions	Alkyl
				VAL109	Hydrophobic interactions	Alkyl
				ASN85	Hydrophobic interactions	Van der Waals
				GLN83	Hydrophobic interactions	Van der Waals
				SER110	Hydrophobic interactions	Van der Waals
				MET112	Hydrophobic interactions	Van der Waals
				LYS113	Hydrophobic interactions	Van der Waals
				ASP80	Hydrophobic interactions	Van der Waals
	(E)-3-Butylidene-4,5-dihydroisobenzofuran-1(3H)-one	5877292	-5.0 kcal/mol	LYS543	Hydrogen bond	Conventional hydrogen bond
				LYS541	Hydrophobic interactions	Pi-alkyl
				LEU494	Hydrophobic interactions	Pi-alkyl
				SER542	Hydrophobic interactions	Van der Waals
				VAL540	Hydrophobic interactions	Van der Waals
				GLY495	Hydrophobic interactions	Van der Waals
				MET374	Hydrophobic interactions	Van der Waals
				ASP80	Hydrophobic interactions	Van der Waals
				GLN83	Hydrophobic interactions	Van der Waals
				THR52	Hydrophobic interactions	Van der Waals
				PHE373	Hydrophobic interactions	Pi-alkyl
				THR53	Hydrophobic interactions	Van der Waals
				PRO54	Hydrogen bond	Carbon–hydrogen bond
	2-(*n*-Morpholino)ethanesulfonic acid	5155249	-4.3 kcal/mol	ASN384	Hydrophobic interactions	Van der Waals
				GLU383	Hydrophobic interactions	Van der Waals
				GLU382	Hydrogen bond	Carbon–hydrogen bond
				LYS282	Hydrophobic interactions	Van der Waals
				LYS281	Hydrophobic interactions	Van der Waals
				HIS387	Other	Pi-sulfur
				ASN309	Hydrophobic interactions	Van der Waals
				LEU279	Hydrophobic interactions	Van der Waals
				MET297	Hydrophobic interactions	Van der Waals
				SER278	Hydrogen bond	Conventional hydrogen bond
				PRO385	Hydrophobic interactions	Van der Waals

CYP2C19 protein (PDB ID: 4GQS)	2-Methoxybenzoic acid, 2,3-dichlorophenyl ester	91514954	-7.0 kcal/mol	ASN107	Hydrogen bond	Carbon–hydrogen bond
				ALA103	Hydrophobic interactions	Pi-alkyl
				LEU102	Hydrophobic interactions	Pi-alkyl
				LEU233	Hydrophobic interactions	Pi-alkyl
				LEU237	Hydrophobic interactions	Pi-alkyl
				LEU237	Hydrophobic interactions	Alkyl
				ALA106	Hydrophobic interactions	Pi-alkyl
				VAL208	Hydrophobic interactions	Van der Waals
				ASN204	Hydrophobic interactions	Van der Waals
				ALA297	Hydrophobic interactions	Pi-alkyl
				LEU366	Hydrophobic interactions	Alkyl
				VAL113	Hydrophobic interactions	Alkyl
				VAL113	Hydrophobic interactions	Pi-alkyl
				PHE476	Hydrophobic interactions	Pi-alkyl
				PHE114	Hydrophobic interactions	Pi-alkyl
				ASP293	Hydrophobic interactions	Van der Waals
				GLY296	Hydrophobic interactions	Van der Waals
	(4-Hydroxy-3,5-dimethylphenyl)(2-methyl-1-benzofuran-3-yl)methanone	66563699	-8.3 kcal/mol	ALA441	Hydrophobic interactions	Pi-alkyl
				THR305	Hydrophobic interactions	Van der Waals
				GLN356	Hydrophobic interactions	Van der Waals
				LEU361	Hydrophobic interactions	Pi-sigma
				PRO427	Hydrophobic interactions	Van der Waals
				PHE428	Hydrogen bond	Carbon–hydrogen bond
				CYS435	Other	Pi-sulfur
				CYS435	Hydrophobic interactions	Alkyl
				CYS435	Hydrophobic interactions	Pi-alkyl
				ILE362	Hydrophobic interactions	Pi-alkyl
				ILE362	Hydrophobic interactions	Alkyl
				LEU366	Hydrophobic interactions	Pi-alkyl
				PHE476	Hydrophobic interactions	Pi-alkyl
				PHE476	Hydrophobic interactions	Alkyl
				VAL113	Hydrophobic interactions	Alkyl
				ALA297	Hydrophobic interactions	Alkyl
				THR301	Hydrophobic interactions	Van der Waals
				GLY298	Hydrophobic interactions	Van der Waals
				THR302	Hydrophobic interactions	Van der Waals

**Table 7:** DFT calculation of the selected compounds.

**Compounds**	**Compound ID**	**LUMO (eV)**	**HOMO (eV)**	**HOMO-LUMO gap (eV)**	**Hardness (eV)**	**Softness (eV)**
(E)-3-Butylidene-4,5-dihydroisobenzofuran-1(3H)-one	CID_5877292	−2.047	−6.092	4.045	2.0225	0.4951
1,2-Hydrazinedicarboxylic acid, dimethyl ester	CID_265934	0.831	−6.249	7.08	3.54	0.2825
1,3-Propanediol, 2-methyl-, dipropanoate	CID_345905	0.267	−7.513	7.71	3.855	0.2594
2-Methoxybenzoic acid, 2,3-dichlorophenyl ester	CID_91514954	−1.499	−6.415	4.916	2.458	0.4068
Bicyclo[7.2.0]undec-4-ene, 4,11,11-trimethyl-8-methylene-,[1R-(1R∗,4Z,9S∗)]	CID_5322111	0.274	−6.154	6.428	3.214	0.3111

## Data Availability

The data that support the findings of this study are available from the corresponding author upon reasonable request.
